# Goitre Treated by Electricity

**Published:** 1890-08-16

**Authors:** 


### GOITRE TREATED BY ELECTRICITY.

Dr. V. Morano, of New South Wales, has recently reported a case of goitre treated by electricity. The patient was a young lady with well marked goitre, the right lobe of the thyroid gland showing the most enlargement. The goitre was of the fibro-cystic variety. Ordinary treatment had no effect. The case was therefore treated by electricity, viz., strong external faradic currents, suddenly interrupted, and electrolysis in alternate sittings. The current for the latter was given by five to eight large Leclanche cells, and was applied by placing the positive pole—a sponge electrode—between the patient's hands, and by inserting in the tumour a small insulated glovers' needle, with which was connected the negative pole. One or two sittings were given per week. A marked improvement, especially in the subjective symptoms, took place from the first séance, while after six sittings the goitre had deminished by one half. Afterwards shrivelling was not so rapid. The author remarks the method is simple and efficacious, the only pain felt being that incidental to the insertion of the needles.

